# Magnetic refrigeration material operating at a full temperature range required for hydrogen liquefaction

**DOI:** 10.1038/s41467-022-29340-2

**Published:** 2022-03-31

**Authors:** Xin Tang, H. Sepehri-Amin, N. Terada, A. Martin-Cid, I. Kurniawan, S. Kobayashi, Y. Kotani, H. Takeya, J. Lai, Y. Matsushita, T. Ohkubo, Y. Miura, T. Nakamura, K. Hono

**Affiliations:** 1 National Institute for Materials Science, Tsukuba, 305-0047 Japan; 2 International Center for Young Scientists, National Institute for Materials Science, Tsukuba, 305-0047 Japan; 3 International Center for Synchrotron Radiation Innovation Smart (SRIS), Tohoku University, Sendai, 980-8577 Japan; 4 Japan Synchrotron Radiation Research Institute, SPring-8, 1-1-1 Kouto, Sayo, 679-5198 Japan; 5 Graduate School of Science and Technology, University of Tsukuba, Tsukuba, 305-8577 Japan

**Keywords:** Materials for energy and catalysis, Materials for devices

## Abstract

Magnetic refrigeration (MR) is a key technique for hydrogen liquefaction. Although the MR has ideally higher performance than the conventional gas compression technique around the hydrogen liquefaction temperature, the lack of MR materials with high magnetic entropy change in a wide temperature range required for the hydrogen liquefaction is a bottle-neck for practical applications of MR cooling systems. Here, we show a series of materials with a giant magnetocaloric effect (MCE) in magnetic entropy change (-∆*S*_*m*_ > 0.2 J cm^−3^K^−1^) in the Er(Ho)Co_2_-based compounds, suitable for operation in the full temperature range required for hydrogen liquefaction (20-77 K). We also demonstrate that the giant MCE becomes reversible, enabling sustainable use of the MR materials, by eliminating the magneto-structural phase transition that leads to deterioration of the MCE. This discovery can lead to the application of Er(Ho)Co_2_-based alloys for the hydrogen liquefaction using MR cooling technology for the future green fuel society.

## Abstract (web summary)

Application of magnetic refrigeration (MR) for hydrogen liquefaction is limited by lack of MR materials with a large magnetocaloric effect (MCE). Here the authors show a series of MR materials with a large and reversible MCE operating at a full temperature range required for hydrogen liquefaction.

## Introduction

**Fig. 1:**
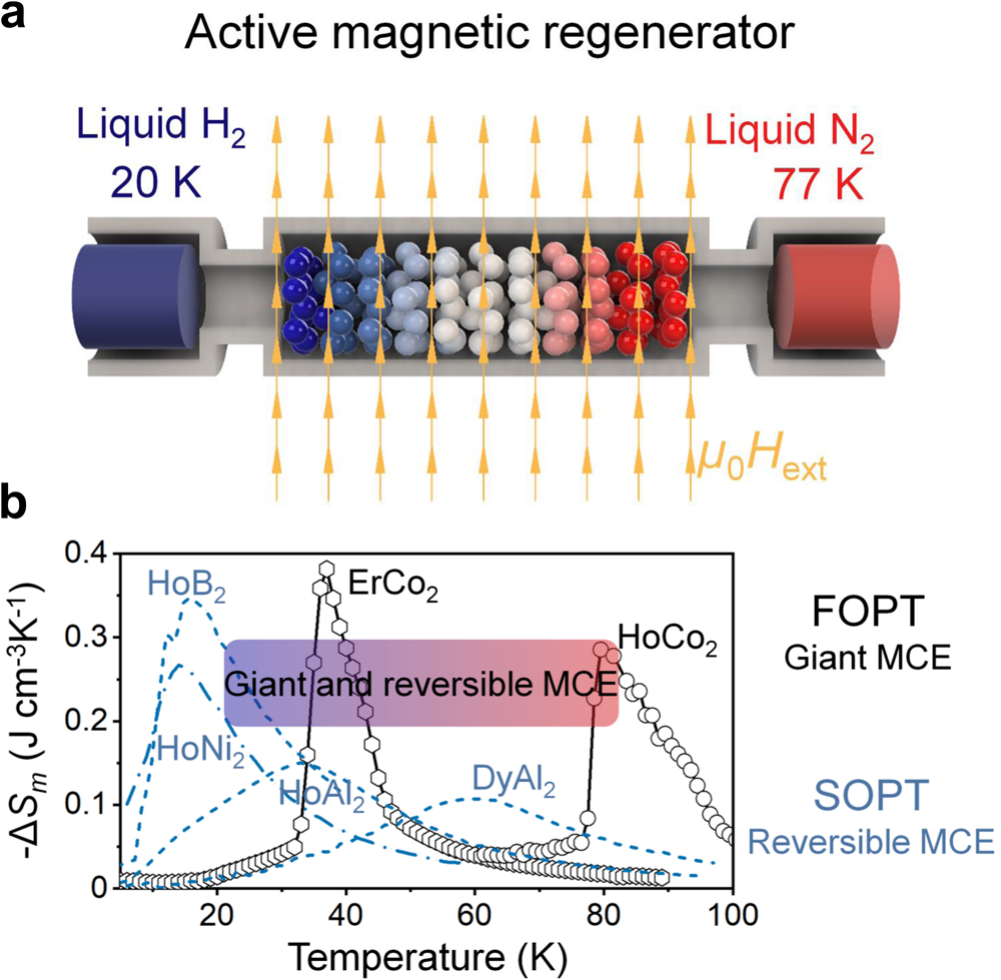
Magnetic refrigeration materials required for hydrogen liquefaction using magnetic refrigeration. **a** Schematic of the active magnetic regenerator for hydrogen liquefaction. A series of magnetic refrigerants with tailored transition temperatures at external field (µ_0_*H*_ext_) are required to cover the large temperature range from the boiling temperature of nitrogen (77 K) to the boiling temperature of hydrogen (20 K). **b** Isothermal magnetic entropy change (−∆*S*_*m*_) as a function of temperature. Second-order magnetic phase transition materials illustrated by dashed blue curves, for example, HoB_2_^20^, HoNi_2_^24^, HoAl_2_^28^, and DyAl_2_^28^ show no hysteresis, a giant MCE only at temperatures below 20 K and small MCE at temperatures above 30 K. First-order magnetic phase transition materials indicated by black curves with symbols exhibit giant magnetocaloric effects (MCEs) (large −∆*S*_*m*_) at a broad temperature range but with thermal hysteresis near the transition temperature, resulting in irreversible giant MCEs. Therefore, giant and reversible MCEs with broad operating temperature window illustrated by gradient colour from red to blue corresponding to the magnetic refrigerants with different transition in (a) from 77 K to 20 K are desirable for active magnetic regenerator.

Magnetic materials undergo isothermal magnetic entropy changes (Δ*S*_*m*_) or adiabatic temperature changes (Δ*T*_ad_) upon the application or removal of an external magnetic field. This phenomenon is known as the magnetocaloric effect (MCE)^1^. Magnetic refrigeration (MR) based on the MCE is considered to be a promising energy-efficient and environmentally benign refrigeration technology^2^. The concept of cooling by adiabatic demagnetisation at the ultra-low temperatures was proposed independently by Debye^3^ and Giauque^4^ based on thermodynamic studies in the late 1920s. This concept was experimentally demonstrated in the early 1930s by Giauque and MacDougall on the adiabatic demagnetisation of Gd_2_(SO_4_)_3_.8H_2_O, leading to attainment of temperatures below 1K^5^. The operation of MR can be also extended to room temperature application using magnetic refrigerants, such as Gd_5_Si_2_Ge_2_^6–8^, (Mn,Fe)_2_P^9^, MnAs^10,11^, Ni-Mn-based Heusler alloy^12,13^ and La(Fe,Si)_13_H^14–16^. Recently, the global demand for the reduction of CO_2_ emission has increased the attention devoted to the use of renewable energy for which hydrogen plays an important role in the so-called decarbonised hydrogen society^17^. In this context, magnetic refrigeration has been demonstrated to be a potential candidate for hydrogen liquefaction and avoidance of hydrogen boil-off during storage^18^. In this approach, hydrogen gas is cooled to ~77 K (boiling temperature of liquid nitrogen) followed by a further decrease in temperature to its liquefaction temperature of 20 K. Because the existing magnetic refrigerant materials cannot maintain a large MCE over the wide temperature span of 77–20 K, a series of refrigerant materials are used in an active magnetic regenerator (AMR) system (Fig. 1a).

The isothermal magnetic entropy change (Δ*S*_*m*_) is given by

1 $$\Delta S_m=\mu_0{\int }_{H}^{0}{\left(\frac{\partial M}{\partial T}\right)}_{H}{dH}$$

and is used to characterise the magnetocaloric response of a magnetic refrigerant, where µ_0_ is the permeability of free space, *M* is the magnetisation, *T* is the temperature, and *H* is the external magnetic field. The maximum value of Δ*S*_*m*_ is achieved near the magnetic transition temperature (*T*_tr_) owing to the large value of $\frac{\partial M}{\partial T}$. HoAl_2_ compound is one of the potential magnetic refrigerant materials for hydrogen liquefaction, which has −△*S*_*m*_ = 0.16 J cm^−3^K^−1^at temperature of 32 K obtained from a single crystal and is called as a material with a giant MCE^1,19^. To realise an efficient AMR system for H_2_ liquefaction applications, a larger magnetic entropy change of −△*S*_*m*_ > 0.2 J cm^−3^K^−1^ covering a broad temperature range of 20–77 K for magnetic refrigerants is needed. Giant MCEs (−△*S*_*m*_ > 0.2J cm^−3^K^−1^) have been realised in Ho-based compounds (Fig. 1b), such as HoB_2_^20^, HoN^21,22^, and HoNi_2_^23,24^ at temperatures below 20 K. These ferro/paramagnetic phase transitions without thermal hysteresis have been classified as second-order magnetic phase transitions (SOPT). SOPT materials without thermal hysteresis intrinsically lead to reversible MCE and mechanical stability during cyclic performance^25,26^, and hence are desirable for practical application. To meet the requirements of AMR, giant MCE must be maintained at temperatures up to 77 K. However, the increase in *T*_tr_ of SOPT materials has been found to be achieved at the expense of |△*S*_*m*_|. For example, |△*S*_*m*_| values of 0.17 J cm^−3^K^−1^ and 0.15 J cm^−3^K^−1^ are obtained at 30 K for the (Gd_*x*_Ho_1-*x*_)B_2_ and HoAl_2_ compounds^27,28^, respectively, and |△*S*_*m*_| decreases to 0.10 J cm^−3^K^−1^ at 60 K for the DyAl_2_ compound^28^. Hence, there are very few refrigerant materials exhibiting a giant MCE of |△*S*_*m*_| > 0.2 J cm^−3^K^−1^ that do not show thermal hysteresis at the temperatures of 30–77 K required for H_2_ liquefaction in AMR systems. By contrast, magnetic refrigerant materials with first-order magnetic phase transition (FOPT) have giant MCEs induced by their magnetostructural phase transitions. For example, in ErCo_2_, a transformation from the paramagnetic phase (cubic) to the ferrimagnetic phase (rhombohedral) results in −△*S*_*m*_ = 0.37 J cm^−3^K^−1^ at 37 K (Fig. 1b)^29^. With Ho substitution for Er, *T*_tr_ can be increased to 80 K while maintaining a giant MCE (−△*S*_*m*_ > 0.2 J cm^−3^K^−1^) in the (Ho_1-*x*_Er_*x*_)Co_2_ compounds^30^. However, the (Ho_1-*x*_Er_*x*_)Co_2_ compounds undergoing FOPT suffer from thermal hysteresis resulting in an irreversible MCE and mechanical instabilities^25,26^, hindering their application for AMR.

In this work, we demonstrate that for (Ho)ErCo_2_ compounds, the substitution of Co by particular 3d metal elements, such as Fe or Fe + Ni, can eliminate the hysteresis by avoiding the structural transformation, while maintaining a giant MCE at the transition temperature. Different from the conventional reports, the narrow operating temperature window is substantially expanded in this work by developing a series of materials with hysteresis-free and tailored transitions at the temperature range of 20–77 K with |△*S*_*m*_| > 0.2 J cm^−3^K^−1^. Thus, this discovery can directly lead to the realisation of the AMR system as a great leap toward the application of magnetic cooling technology for hydrogen liquefaction.

## Results

### Tuning the transition temperature and thermal hysteresis

**Fig. 2:**
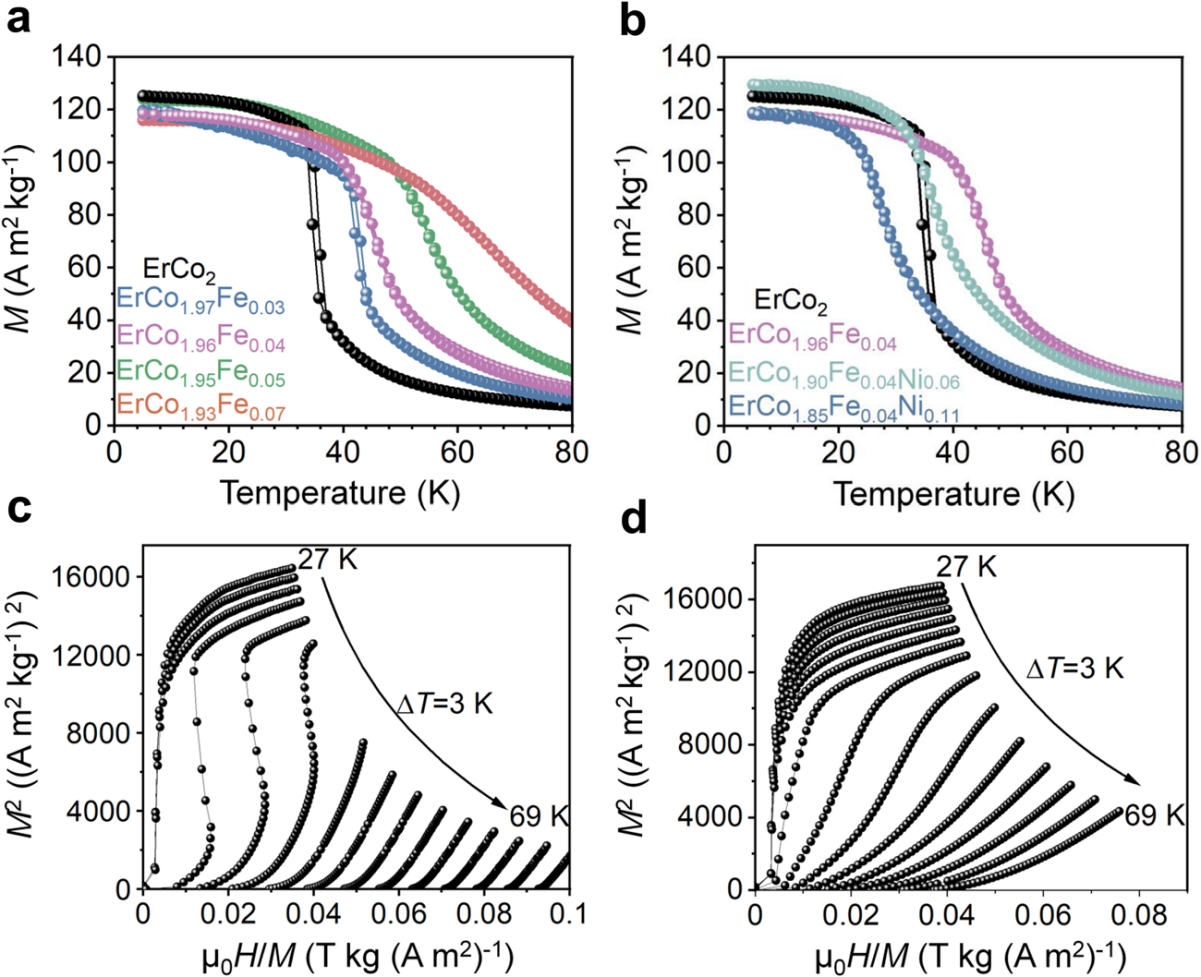
Tuning the transition temperature, thermal hysteresis, and order of magnetic phase transition in an ErCo_2_-based system. **a**, **b** The Magnetisation (*M*) is plotted versus temperature at an applied magnetic field of 1T showing that the transition temperature can be tuned to higher temperatures (**a**) and to lower temperatures (**b**), while eliminating the hysteresis by substituting Co by Fe and Fe + Ni in the ErCo_2_-based system. The colours of the curves in **a**, **b** correspond to the colour of listed alloy compositions. **c**, **d** The Arrott plots measured from 27 K to 69 K at a temperature step (△*T*) of 3 K for **c** ErCo_2_ and **d** ErCo_1.95_Fe_0.05_ alloy show the changes of the phase transition from first-order to second-order, respectively.

Magnetisation as a function of temperature *M*(T) at a magnetic field of 1T is plotted for ErCo_2_ and Er(Co,Fe,Ni)_2_-based alloys (Fig. 2a, b). The ErCo_2_ alloy itself undergoes the paramagnetic/ferrimagnetic transition at *T*_*C*_ = 37 K that accompanies a magnetostructural phase transition that gives rise to an abrupt change in the magnetisation, and correspondingly induces a giant magnetic entropy change of 0.37 J cm^−3^K^−1^ (supplementary Fig. 1). This comes at the cost of thermal hysteresis of 2 K (Fig. 2a), hindering the reversibility of MCE and the practical application of these materials. Figure 2a, b show that the partial substitution of Co with Fe or Fe + Ni solves this problem. The elimination of the thermal hysteresis was successfully achieved in the ErCo_1.96_Fe_0.04_ alloy with a broad transition temperature span, showing the features of SOPT. Further increase of Fe substitution for Co leads to less sharp transitions observed from the *M*-*T* curves and a reduction of magnetic entropy changes, as shown in supplementary Fig. 1. Tunable transition temperature is another requirement of magnetocaloric materials for AMR systems. As observed from Fig. 2a, the transition temperature increased from 37 K to 62 K when *x* increased to 0.07 in the ErCo_2−*x*_Fe_*x*_ alloy while hysteresis was eliminated. The transition temperature can also be tuned toward a lower temperature (28 K) when Ni is alloyed in the ErCo_1.96_Fe_0.04_ alloy, while maintaining the hysteresis-free state, as shown in Fig. 2b. Refrigeration capacity (RC), defined as the integrated area under the -Δ*S*_*m*_(*T*) curve at the peak’s half value is another important parameter that is a measure for the amount of heat transfer from the cold to the hot reservoirs in a single ideal MR cycle. The RC is improved from 2.2 J cm^−3^ for ErCo_2_ to 2.42 J cm^−3^ in the case of the ErCo_1.96_Fe_0.04_ alloy. With a further increase in Fe content, a magnetic entropy change of 0.17 J cm^−3^K^−1^ at 57 K can be obtained for the ErCo_1.95_Fe_0.05_ alloy with an enhanced RC of 2.95 J cm^−3^. Although conventionally, a larger RC is achieved at the expense of the peak value of -Δ*S*_*m*_, making the use of magnetic refrigerants with SOPT less attractive, the |Δ*S*_*m*_| value for the ErCo_2-*x*_Fe_*x*_ (*x* ≥ 0.04) alloys developed in this study induces the largest entropy change of 0.21 J cm^−3^K^−1^ within the temperature range of 30–77 K compared to all known refrigerant materials without hysteresis reported to date^1,21–24,27,28,31,32^, Arrott plots were employed (Fig. 2c, d) to determine the order of the magnetic phase transitions. A negative slope and reflection points near the transition temperature were observed for the ErCo_2_ alloy, revealing a typical FOPT based on the Banerjee criterion^33^. The negative slope was strongly suppressed, and a characteristic of SOPT was observed for the compositionally-modified ErCo_1.95_Fe_0.05_ alloy.

### Characterisation of phase transitions

**Fig. 3:**
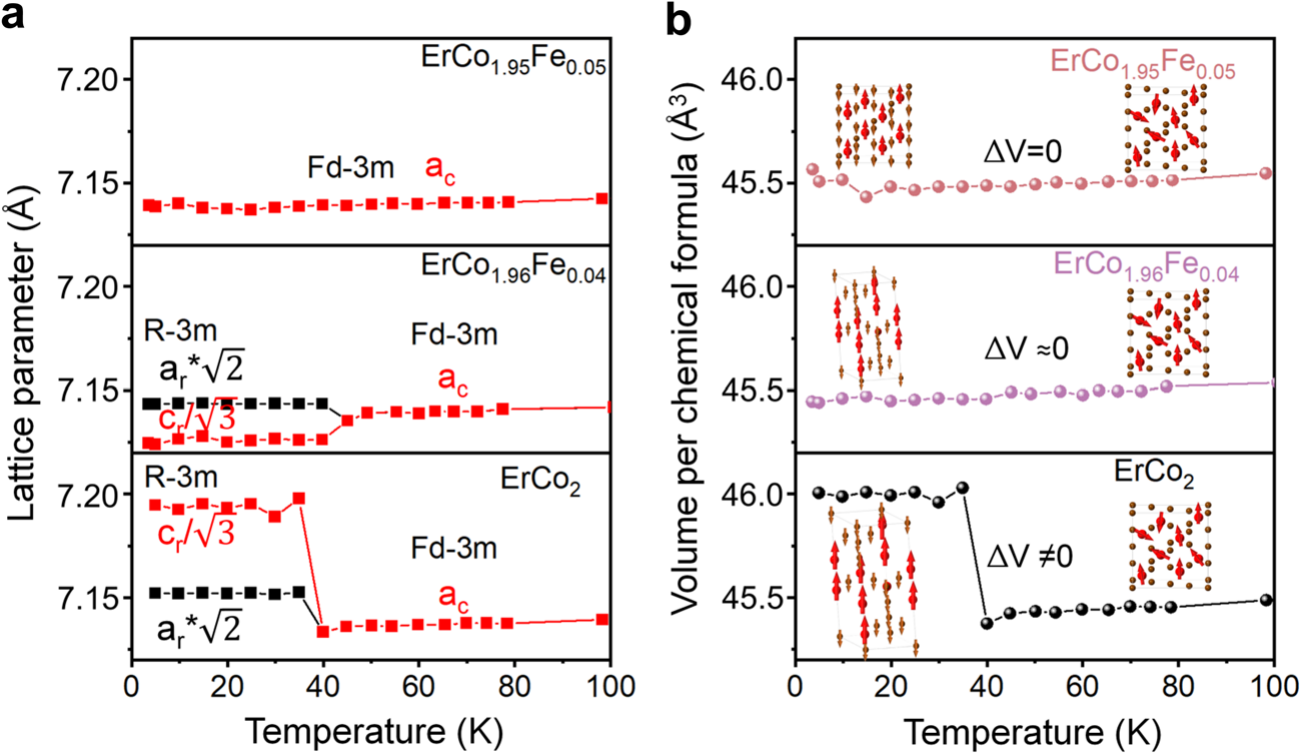
Analysis of the lattice constant and crystal volume change around the transition temperature range using cryogenic x-ray diffraction (XRD). **a** Temperature dependence of the lattice parameters for ErCo_2_-based alloys. The lattice parameters are obtained from the Rietveld refinement. The lattice parameters of the rhombohedral phase are plotted for $\sqrt{2}{a}_{r}$ (black) and ${c}_{r}/\sqrt{3}$ (red) to compare directly with the lattice parameter of the cubic phase. **b** Temperature-dependent volume per chemical formula for ErCo_2_ (black), ErCo_1.96_Fe_0.04_ (purple) and ErCo_1.95_Fe_0.05_ (red) alloys, the crystal structure change upon transition is also illustrated by a unit cell (red and brown spheres represent Er and Co atoms, respectively, and red and brown arrows represent the magnetic moments of the Er and Co atoms, respectively).

Although the Arrott plots qualitatively show the nature of the magnetic phase transition, the latter can be investigated further by tracking the change in the crystal lattice during the phase transition. An unambiguous assessment of the phase transition was carried out using in-situ XRD measurement under cryogenic temperatures. The details of the determination of the phase transition by XRD are shown in the supplementary note 1. The change in the lattice spacing upon cooling from 100 to 5 K is plotted in Fig. 3a. Because the quantities $\sqrt{2}{a}_{r}$ and ${c}_{r}/\sqrt{3}$ of the lattice parameters for the rhombohedral phase are equivalent to the *a*_c_ of the cubic lattice,$\,\sqrt{2}{a}_{r}$ and ${c}_{r}/\sqrt{3}$ for the rhombohedral phase are plotted to compare directly with the lattice parameter of the cubic phase. A step jump in the lattice spacing in ErCo_2_ was observed near the transition temperature for the ErCo_2_ alloy. With Fe substitution for Co in the ErCo_1.96_Fe_0.04_ alloy, the lattice constant change was substantially reduced, even though a cubic/rhombohedral crystal structure change was observed. This result clearly shows that the addition of Fe can suppress the large volume change that occurs in the magnetostructural phase transition in ErCo_2_ at *T*_tr_. A further increase in Fe content in the ErCo_1.95_Fe_0.05_ alloy leads to no changes in the lattice spacing or volume per chemical formula caused by the structural transition near *T*_tr_, indicating the realisation of SOPT. In other words, this work demonstrates a concept that the substitution of a 3*d* metal element, such as Fe for Co in the ErCo_2_-based Laves phase eliminates the structural phase transition, thereby reducing the thermal hysteresis to 0 K.

**Fig. 4:**
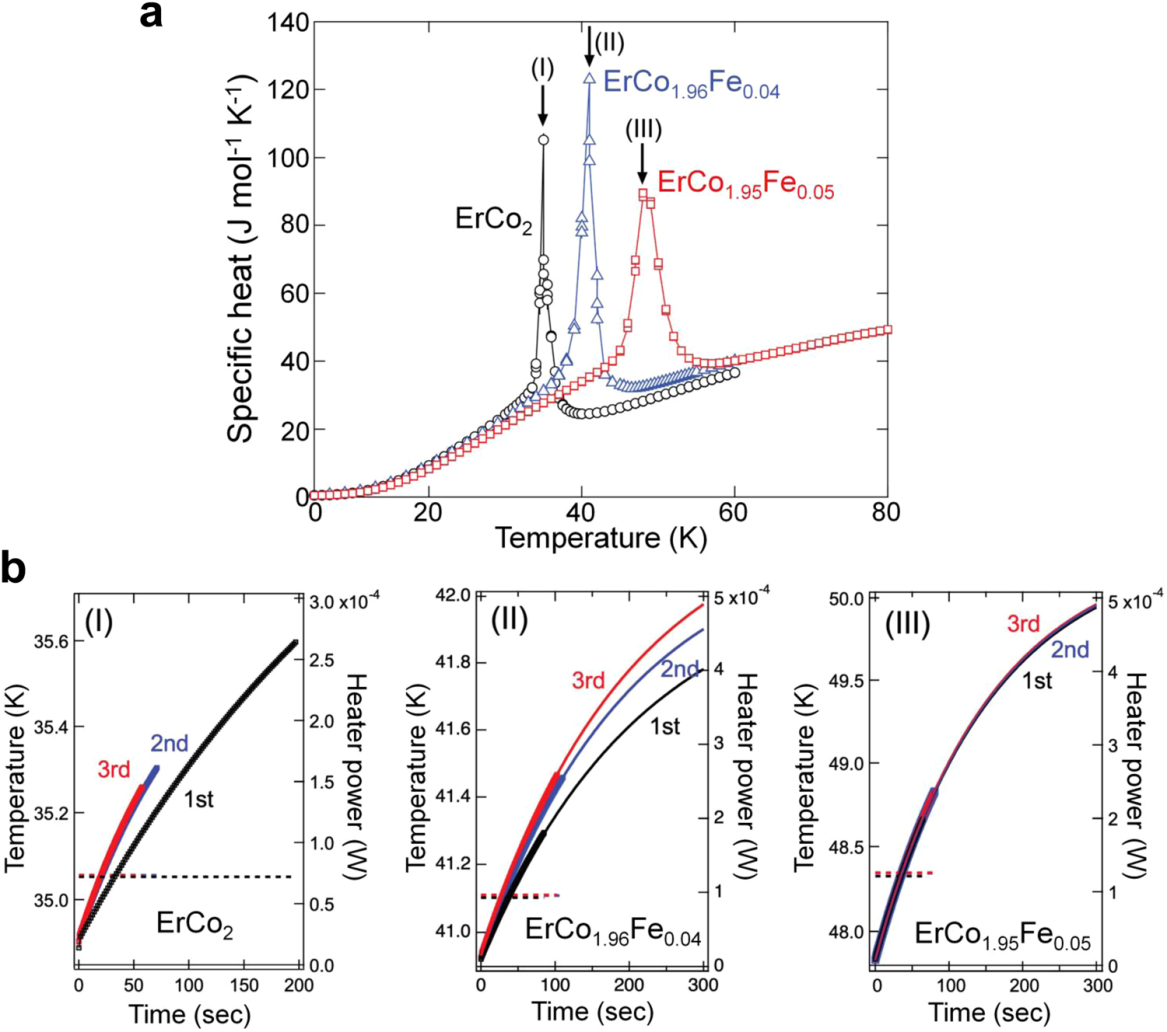
Analysis of the latent heat involved with first-order phase transition. **a** Temperature dependence of the specific heat measured by the thermal relaxation method for ErCo_2_ (black), ErCo_1.96_Fe_0.04_ (blue) and ErCo_1.95_Fe_0.05_ (red) alloys. **b** Time evolution of the sample temperature obtained using the relaxation method at the temperature where each sample shows a peak anomaly indicated by arrows (I), (II), and (III) in **a**. The dashed lines denote heat power for each measurement (1^st^ (black), 2^nd^ (blue) and 3^rd^ (red) measurements) in **b**.

A further characterisation of the nature of the phase transitions in Er(Co_1-*x*_Fe_*x*_)_2_ was performed by measuring the specific heat, $C$, using the thermal relaxation method, the details of which are described in the supplementary note 2. In Fig. 4a, the specific heat values given by three measurements are the same in each temperature for the three samples (ErCo_2_, ErCo_1.96_Fe_0.04_ and ErCo_1.95_Fe_0.05_ alloys), within the experimental accuracy, apart from the temperatures close to the phase transition temperatures. Here, we analysed the dependence of the time evolution of the sample temperature on the phase transition temperatures for the three samples shown in Fig. 4b. For ErCo_2_, a significant difference in *T*(*t*) was found at the phase transition temperature of ~35 K, as indicated by arrow (I) in Fig. 4a. The temperature rise during the first run is significantly suppressed by the existing latent heat, compared to those in the second and third runs. The strong thermal hysteretic behaviour observed in Fig. 2 for the phase transition of ErCo_2_ corresponds to the FOPT behaviour. For sample II (ErCo_1.96_Fe_0.04_), a similar behaviour was observed (Fig. 4b), indicating that the phase transition is still first-order. It is clear that the $\Delta {T}_{{\max }}$ value in the first measurement is lower than those in the second and third measurements. On the other hand, the hysteretic behaviour was not observed within the experimental accuracy in sample III (ErCo_1.95_Fe_0.05_) as shown in Fig. 4b. Thus, we conclude that the phase transitions for ErCo_2_ and ErCo_1.96_Fe_0.04_ are FOPT, while the ErCo_1.95_Fe_0.05_ sample does not show FOPT behaviour, which is consistent with that observed by in-situ XRD shown in Fig. 3.

### Origin of large magnetocaloric effect

**Fig. 5:**
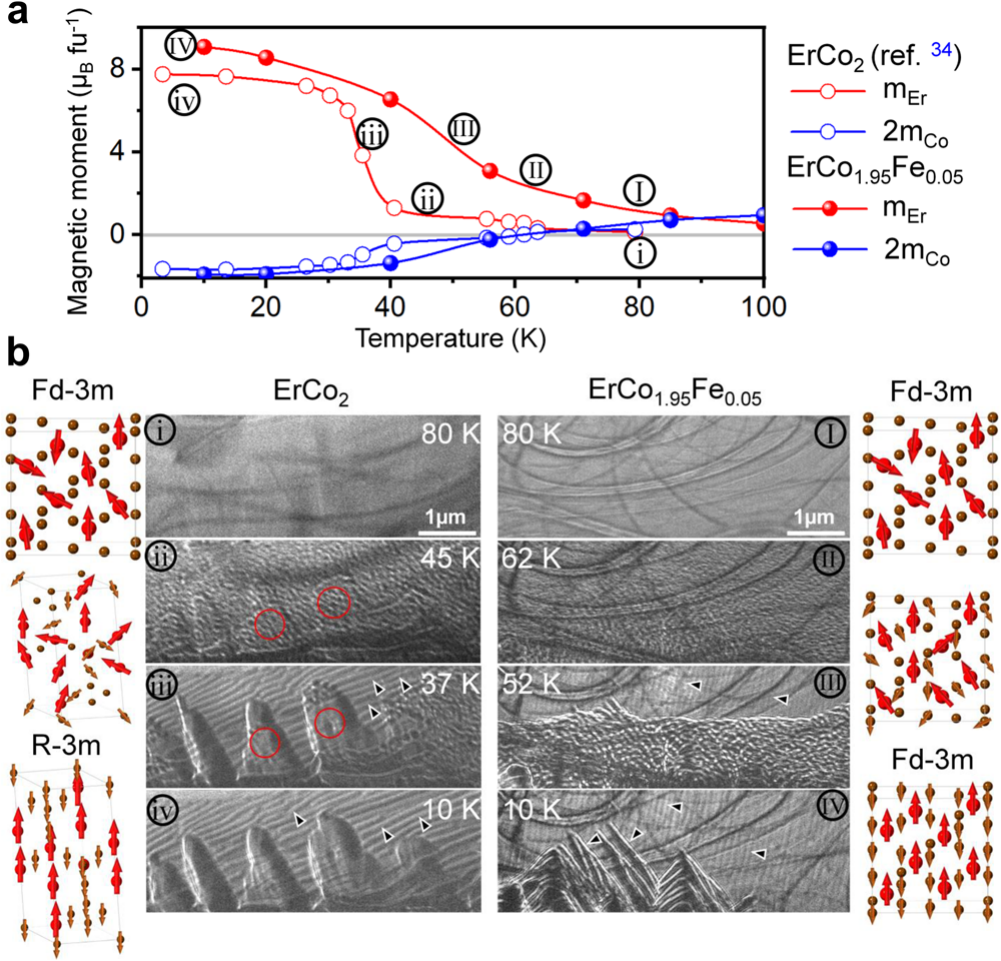
Magnetism and magnetic structure for ErCo_2_-based alloys. **a** Temperature-dependent magnetic moments for Er (red) and Co (blue) in ErCo_2_^34^, and ErCo_1.95_Fe_0.05_ alloys. **b** Magnetic domain evolution in ErCo_2_-based alloys examined using cryogenic Lorentz microscopy in the Fresnel mode. The black arrow heads show the magnetic domains and red circles illustrate the mountain-like contrasts caused by strain; the crystal structure change upon transition is also shown by a unit cell (red and brown spheres for Er and Co atoms, respectively, and red and brown arrows for the magnetic moments of Er and Co, respectively).

Figure 5a shows the results of the specific magnetometric measurements of Er and Co probed by X-ray magnetic circular dichroism (XMCD). Note that the data for the ErCo_2_ alloy were adopted from reference^34^. The Co magnetisation at low temperatures (<10 K) remains opposite to the Er magnetisation after alloying with Fe, thus revealing that the ferrimagnetic behaviour is not changed by the substitution of Co with Fe. Unlike for ErCo_2_ alloy that exhibits a sharp change in the magnetic moment of Er at *T*_tr_, the change in the magnetic moment of Er is more gradual around *T*_tr_, which is consistent with the *M–T* curves presented in Fig. 2. The evolution of the magnetic moments of Er and Co can be directly seen from the XMCD signal shown in the Supplementary Fig. 2. In the XMCD signal of the Co L_2,3_ edges, an inversion of the edges appears at the temperatures above 56 K, corresponding to the change in the sign of the magnetic moment of Co. For the Er M_4,5_ XMCD spectra, the intensity of the signal decreases as the temperature increases. In-situ Lorentz microscopy observation in the Fresnel mode was employed to understand the evolution of magnetic domains during cooling. Figure 5b shows the selected micrographs in the cooling process at four different temperatures. These are also marked as i–iv and I–IV in Fig. 5a for ErCo_2_ and ErCo_1.95_Fe_0.05_ alloys, respectively. For both samples, there is no contrast in the paramagnetic state. The XMCD result in Fig. 5a shows that the magnetic moment of Er atoms increases upon cooling of the ErCo_2_ sample via the magnetic momentum alignment, while the net magnetic moment of Co remains almost zero. This results in the formation of fine and maze-like magnetic domain structures, as shown in ii in Fig. 5b. Note that close to the *T*_tr_ of ErCo_2_, a mountain-like contrast marked by red circles appears originating from a large strain caused by the cubic/rhombohedral structural transformation. The change in the magnetic domain structure upon cooling to 37 K is due to a substantial increase in the magnetic moment of Er by formation of ferrimagnetic phase, as shown in Fig. 5a. Note that unlike the ErCo_2_ sample, no distinct strain contours were observed in the ErCo_1.95_Fe_0.05_ sample because the cubic structure was preserved at the temperatures below 52 K in the latter case. The observed transition in the shape of the magnetic domains from II to IV, *i. e*. fine maze-like domain patterns in II, strip-like domain patterns in IV, and their mixture in III (indicated by black arrow heads), are due to the gradual increase in the magnetic moment of Er upon a decrease in temperature without existence of any strain contour. Note that the ErCo_1.95_Fe_0.05_ phase is in its ferrimagnetic state at the temperature of 10 K.

The giant MCE in the ErCo_2_ alloy originates from the magnetostructural phase transition and itinerant electron metamagnetism (IEM). The latter was proposed by Wohlfarth and Rhodes^35^. According to the proposed concept, the creation of a magnetic moment in Co is a consequence of the IEM induced by localised ferromagnetic ordering of Er moments that induces a large exchange field at the transition temperature. This can be observed in Fig. 5a, wherein Co has nearly zero magnetisation in the paramagnetic state, while it antiferromagnetically couples with Er at temperatures below 37 K. In this study, we found SOPT with the substitution of Fe for Co in the ErCo_1.95_Fe_0.05_ alloy, in which no structural transformation was observed, while a giant MCE was induced. However, based on the XMCD results, the magnetic moment of Co at temperatures below *T*_tr_ remains comparable in ErCo_2_ and ErCo_1.95_Fe_0.05_ alloys, suggesting that IEM is preserved upon a small substitution of Fe for Co. In addition to the structural transition, any change in the density of states at the Fermi level between these two alloys influences the IEM in Fe-doped alloys^36,37^. We calculated the density of states using density functional theory (DFT) using experimental lattice constants (Supplementary Fig. 3). Trace amounts of Fe substitution for Co have negligible influence on the electronic structure around the Fermi level compared with the cubic/rhombohedral ErCo_2_. This implies that the instability of the 3d sublattice magnetism and IEM is preserved for the ErCo_1.95_Fe_0.05_ alloy, which is consistent with the experimental data obtained by XMCD (Fig. 5a). This observation is in contrast to the conventional belief that FOPT results from IEM^36,37^. Here, we demonstrate that by eliminating the structural phase transition, thermal hysteresis can be eliminated, while the giant MCE can still be achieved due to the preserved IEM in the ErCo_1.95_Fe_0.05_ alloy.

## Discussion

**Fig. 6:**
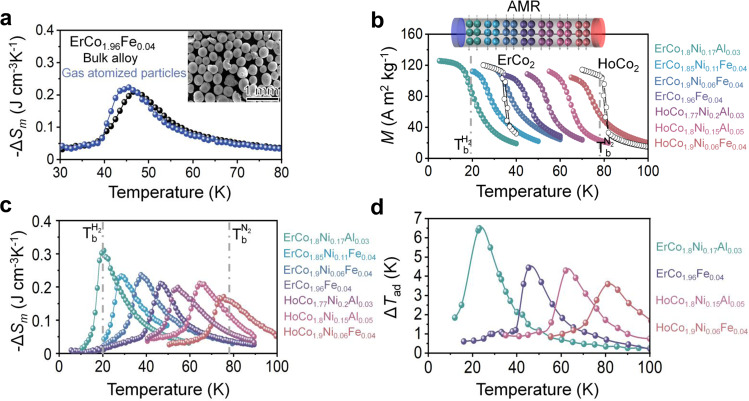
Magnetocaloric effects in compositionally engineered (Ho)ErCo_2_-based alloys. **a** Comparison of the temperature-dependent magnetic entropy change (−Δ*S*_*m*_(*T*)) of a bulk sample (black) and gas-atomised (blue) ErCo_1.96_Fe_0.04_ particle. The inset shows a scanning electron microscopy (SEM) image of a gas-atomised sample. **b** Temperature-dependent magnetisation *M*(*T*) measurements of the cooling and heating branches from 5 K to 100 K at 1 K intervals (µ_0_*H* = 1*T*) for a series of magnetic refrigerants with tailored transition temperatures, the spherical particles in AMR with different colours correspond to different magnetic refrigerants with different transition temperatures in **b**. **c** Temperature-dependent magnetic entropy change −Δ*S*_*m*_(*T*) curves of magnetic refrigerants with tailored transition temperatures covering the large temperature window from 20 K to 77 K. **d** Adiabatic temperature change (Δ*T*_ad_) at the field change (Δ*H*) of 5T of the selected magnetic refrigerants developed in this work at their transition temperatures. The selected colours of the curves in **b**–**d** correspond to the colour of listed alloy compositions.

In practical applications, the surface/volume ratio of the magnetic refrigerants should be increased to achieve better heat exchange between the refrigerants and heat-exchanger fluid. Hence, spherical particles are desired. We employed a gas atomisation method and obtained spherical particles with an average particle diameter in the range of 210–350 μm. An example of spherical particles of the ErCo_1.96_Fe_0.04_ alloy is shown in the inset of Fig. 6a. The optimal annealing of the gas-atomised powders (supplementary Fig. 4) led to the realisation of a −Δ*S*_*m*_ value comparable to that of the bulk sample (Fig. 6a). To develop magnetic refrigeration materials suitable for a broad temperature range of 77–20 K, we further investigated different 3d metal dopants to ErCo_2_ and HoCo_2_-based compounds. We substituted Ni, Al, Fe, and their combinations for Co in Er(Ho)Co_2_ alloys. Figure 6b shows that hysteresis can be eliminated in conventional FOPT-type ErCo_2_ and HoCo_2_ alloys by the doping, and the transition temperature can be tuned from 20 K to 77 K. These changes enable the values of −Δ*S*_*m*_ > 0.2 J cm^−3^K^−1^ to be achieved in the range from 20 to 77 K to cover the required temperature range for hydrogen liquefaction (Fig. 6c). The magnetic refrigerants developed in this study yielded significantly larger entropy changes than those of HoNi_2_^24^, HoAl_2_^28^, REMn_2_X_2_^38–40^, RECo_2_Mn_x_^41^ and DyAl_2_^28^ for cryogenic applications, particularly in the temperature range from 30 K to 77 K. For example, the −Δ*S*_*m*_ values for the ErCo_1.85_Ni_0.11_Fe_0.04_ and HoCo_1.8_Ni_0.15_Al_0.05_ alloys are 55% and 93 % larger than those of HoAl_2_ and DyAl_2_ at the same transition temperatures. Further characterisation of Δ*T*_ad_ of magnetic refrigerants is depicted in Fig. 6d; above 4 K, Δ*T*_ad_ can be achieved for the magnetic refrigerants developed in this work that can cover the application temperature range from 20 to 77 K for hydrogen liquefaction.

Hysteresis is a widespread phenomenon resulting from the nature of the first-order phase transition in magnetic refrigerants that leads to the irreversibility of the magnetocaloric response. Herein, we demonstrate that the elimination of hysteresis can be realised by avoiding the crystal structure change across the phase transition, specifically by alloying with 3d elements, while maintaining the giant magnetocaloric effect comparable to that of a conventional FOPT Er(Ho)Co_2_ alloy. The role of Fe in suppressing the structural transformation upon phase transition is due to the change from FOPT to SOPT as revealed by in-situ XRD, latent heat measurement, and Lorentz TEM for ErCo_1.95_Fe_0.05_. Moreover, the giant MCE was maintained for SOPT because of the preservation of IEM as found by XMCD experiments and DFT calculations. The Er(Ho)Co_2_-based alloys developed in this study have numerous merits, such as significantly larger magnetic entropy changes compared with those of known materials in the temperature range of 20–77 K, a wide operating temperature window due to the stacking of the refrigerants covering the range of application temperatures for hydrogen liquefaction, easy fabrication, and stable magnetocaloric properties of gas-atomised particles. This may lead to the application of Er(Ho)Co_2_-based alloys for hydrogen liquefaction using an active magnetic regenerator. Moreover, the approach for achieving giant reversible MCE in this study is also expected to be applicable for eliminating the detrimental hysteresis of magnetic refrigerants with FOPT for applications near room temperature, such as Gd_5_Si_2_Ge_2_^6–8^, (MnFe)_2_P^9^ and La(FeSi)_13_H^14,15^.

## Methods

### Sample preparation

Polycrystalline (Ho)ErCo_2_-based samples were prepared by arc melting pure constituent elements in an Ar atmosphere, with 2–5 wt.% extra Er and Ho added to compensate the evaporation during sample preparation. The ingots were remelted four times after flipping over to achieve better homogeneity. Thereafter, the ingots were sealed in quartz in an argon atmosphere and annealed for 50 h at 1273 K. The phase constituents and crystal structure were examined by XRD (Rigaku SmartLab 9 kW) with Cu Kα_1_ radiation in the temperature range of 5–300 K. Thermomagnetic measurements were conducted using a superconducting quantum interference device magnetometer (SQUID-VSM).

### Thermomagnetic measurements

To directly measure the adiabatic temperature change, a zirconium oxynitride thin-film CernoxTM thermometer (CX-SD, Lake Shore Cryotronics) was placed on the large surface of a cubic-shaped sample and fixed by thin copper wires. The sample assembly was inserted into the quantum design physical property measurement system (PPMS). The temperature and magnetic field were controlled by PPMS. The sample space was pumped by a cryopump, and the pressure was maintained below 10^−4 ^Torr to reach adiabatic conditions. The thermal relaxation method for latent heat measurements was implemented in the physical properties measurement system (PPMS) manufactured by Quantum Design. To evaluate the time evolution of sample temperature during measurement, the raw data were extracted for each measurement.

### Cryogenic microstructure characterisations

Cryogenic Lorentz microscopy was conducted using ultra-high-voltage (1.2 MV) Hitachi-TEM instrument. The temperature of the TEM specimen was reduced to ~10 K in a cryostat TEM sample holder using liquid helium. The specimens for the TEM analysis were prepared by an FEI Helios G4-UX dual-beam system using the lift-out method. The soft XMCD spectra at the Er M_4,5_ and Co L_2,3_ edges were recorded using the total electron yield method at the BL25SU beamline of SPring-8. The XMCD signal (µ_m_) was obtained as ${{{{{{\rm{\mu }}}}}}}_{{{{{{\rm{m}}}}}}}=({{{{{{\rm{\mu }}}}}}}_{{{{{{\rm{l}}}}}}-}+{{{{{{\rm{\mu }}}}}}}_{{{{{{\rm{r}}}}}}+})-({{{{{{\rm{\mu }}}}}}}_{{{{{{\rm{l}}}}}}+}+{{{{{{\rm{\mu }}}}}}}_{{{{{{\rm{r}}}}}}-})$, where ${{{{{{\rm{\mu }}}}}}}_{{{{{{\rm{r}}}}}}}$ and ${{{{{{\rm{\mu }}}}}}}_{{{{{{\rm{l}}}}}}}$ represent the X-ray absorption spectrum (XAS) for the helicity plus (${{{{{{\rm{h}}}}}}}_{+}$) and minus (${{{{{{\rm{h}}}}}}}_{-}$) of soft X-rays, respectively, and ${{{{{{\rm{\mu }}}}}}}_{+}$ and ${{{{{{\rm{\mu }}}}}}}_{-}$ respectively represent XAS for a positive and negative applied field with intensity equal to 1.9 T. The degree of circular polarisation was previously estimated as 0.96 at 400 eV^42^ and was expected to be similar or larger in the energy region used in the present work. The angle between the magnetic field and the incident X-ray beam was 10°(ref. ^43^). The rod-shaped sample was fractured in the ultra-high vacuum chamber of the XMCD apparatus with the vacuum level of <5 × 10^−7^ Pa to obtain a fresh surface for the XMCD experiments. The samples were measured at different temperatures between 10 and 100 K. The magnetic moments were calculated using the magneto-optical sum rule analysis for XMCD^44–46^ by taking into consideration the spin-correction factor necessary to apply the sum rules for rare earths^47^.

### First-principles calculations

Density functional theory (DFT) calculations were performed using the full-potential linearised augmented plane wave (FLAPW) method as implemented in the WIEN2K code^48^. The exchange-correlation interaction was approximated using the Perdew–Burke–Ernzerhof (PBE)^49^ formulation based on the generalised gradient approximation (GGA). The k-point sampling of the Brillouin zone was performed using 11 × 11 × 11 k-mesh grids. The spin-orbit coupling (SOC) was included in the calculations self-consistently. The localised 4 f electrons of Er were treated based on the implementation of the Hubbard’s *U* parameter using the DFT + *U* method with *U* = 10 eV and *J* = 0.75 eV, including the orbital polarisation. The experimental lattice constants for the rhombohedral and cubic structures were used, and Fe doping in ErCo_1.95_Fe_0.05_ was simulated using the virtual crystal approximation^50^. The ferrimagnetic behaviour and 6 μB orbital moments for Er were confirmed in all systems.

## Supplementary information

Supplementary Information

## Data Availability

All data are available within the Article and Supplementary Files, or available from the corresponding authors upon reasonable request. Source data are provided with this paper.

## Source data

Source Data
