# Supplementary material for: Magnetic refrigeration material operating at a full temperature range required for hydrogen liquefaction
Source: Nat Commun. 2022 Mar 31;13:1817. doi: 10.1038/s41467-022-29340-2 (PMC8971455; doi:10.1038/s41467-022-29340-2)
Supplement: Supplementary file 1 — Supplementary Information [file 41467_2022_29340_MOESM1_ESM.pdf]

Supplementary information for

Magnetic refrigeration material operating at a full temperature range required for hydrogen liquefaction

Xin Tang[1,2], H. Sepehri-Amin[1,3*], N. Terada[1], A. Martin-Cid[4], I. Kurniawan[1], S. Kobayashi[4], Y. Kotani[4], H. Takeya[1], J. Lai[1], Y. Matsushita[1], T. Ohkubo[1], Y. Miura[1], T. Nakamura[1,3,4], K. Hono[1]

[1]National Institute for Materials Science, Tsukuba, 305-0047, Japan

[2]International Center for Young Scientists, National Institute for Materials Science, Tsukuba 305-0047, Japan

[3]International Center for Synchrotron Radiation Innovation Smart (SRIS), Tohoku University, Sendai 980-8577, Japan

[4]Japan Synchrotron Radiation Research Institute, SPring-8, 1-1-1 Kouto, Sayo 679-5198, Japan

* Corresponding author. E-mail: h.sepehriamin@nims.go.jp

The PDF file includes:

Supplementary Figure 1: Magnetocaloric response for $ErCo_2$-based alloys.

Supplementary Figure 2: Evolutions of X-ray magnetic circular dichroism (XMCD) spectra with temperature.

Supplementary Figure 3: First-principle calculations.

Supplementary Figure 4: Optimal magnetocaloric properties of as-gas atomised particles.

Supplementary Note 1: Determination of phase transition by X-ray diffraction pattern.
Supplementary Note 2: Specific heat measurements.

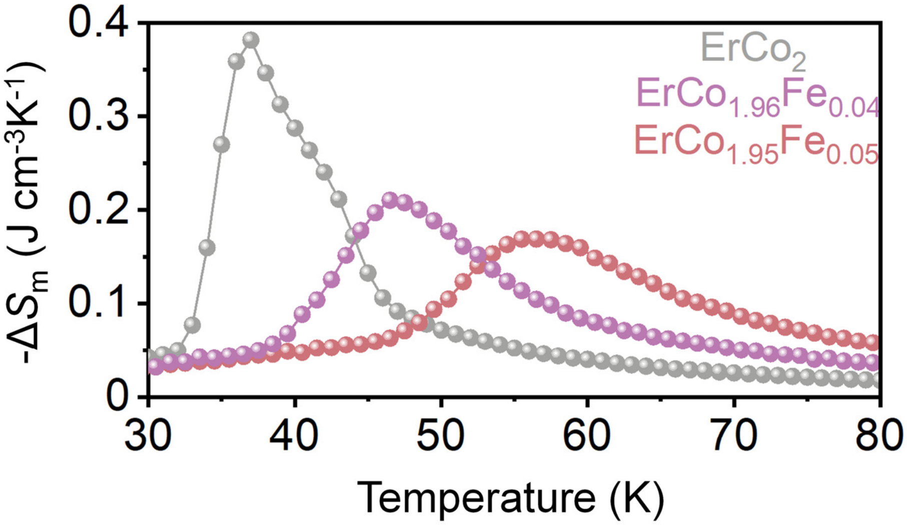

Supplementary Fig. 1. **Magnetocaloric response for $ErCo_2$-based alloys**. Isothermal magnetic entropy change ($-\Delta S_m$) as a function of temperature, $-\Delta S_m(T)$ measured for magnetic fields with strengths in the range of 0–5 T at field steps of 0.5 T and temperature intervals of 1 K, calculated based on Maxwell's equation, from $ErCo_2$ (grey), $ErCo_{1.96}Fe_{0.04}$ (pink), and $ErCo_{1.95}Fe_{0.05}$ (orange) alloys.

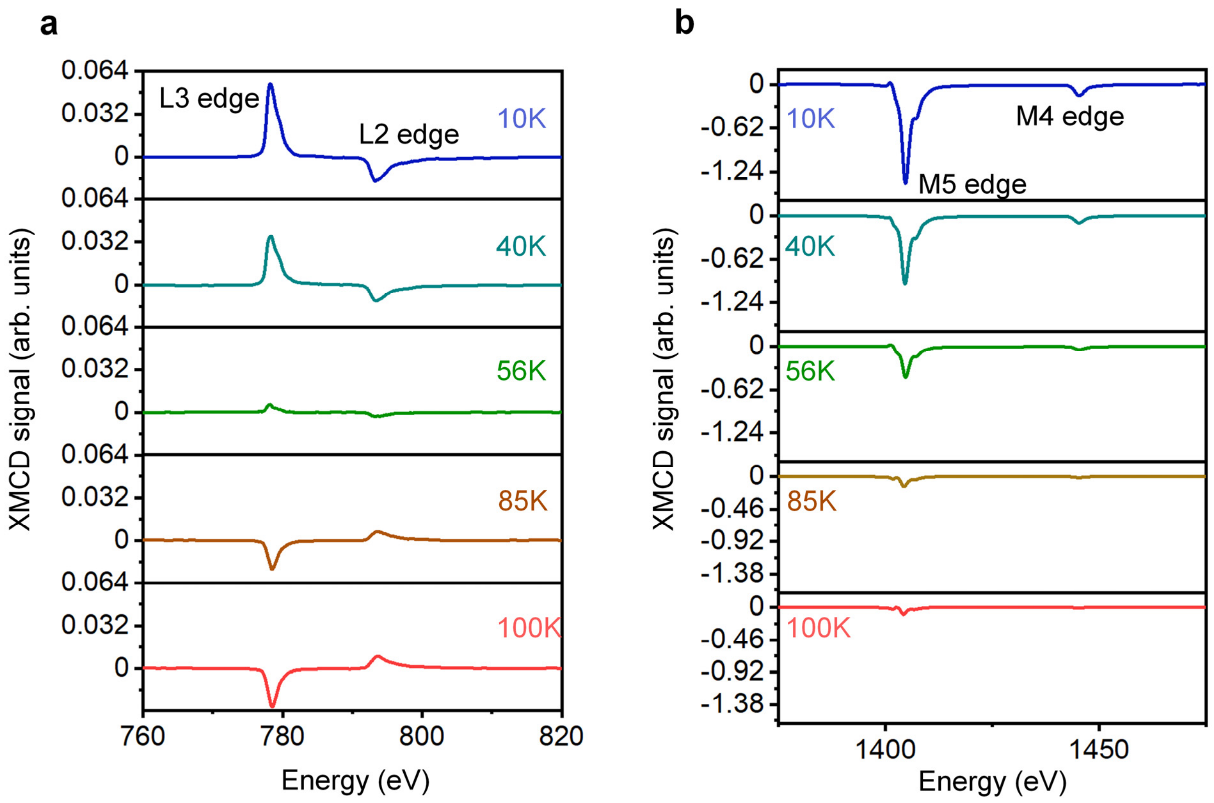

Supplementary Fig. 2. **Evolutions of X-ray magnetic circular dichroism (XMCD) spectra with temperature**. **a** Co $L_{2,3}$ absorption edges. **b** Er $M_{4,5}$ absorption edges obtained from the $ErCo_{1.95}Fe_{0.05}$ sample. The shape of the Co XMCD spectra is inverted for temperatures above 56 K, showing the inversion of the magnetic moment of this element.

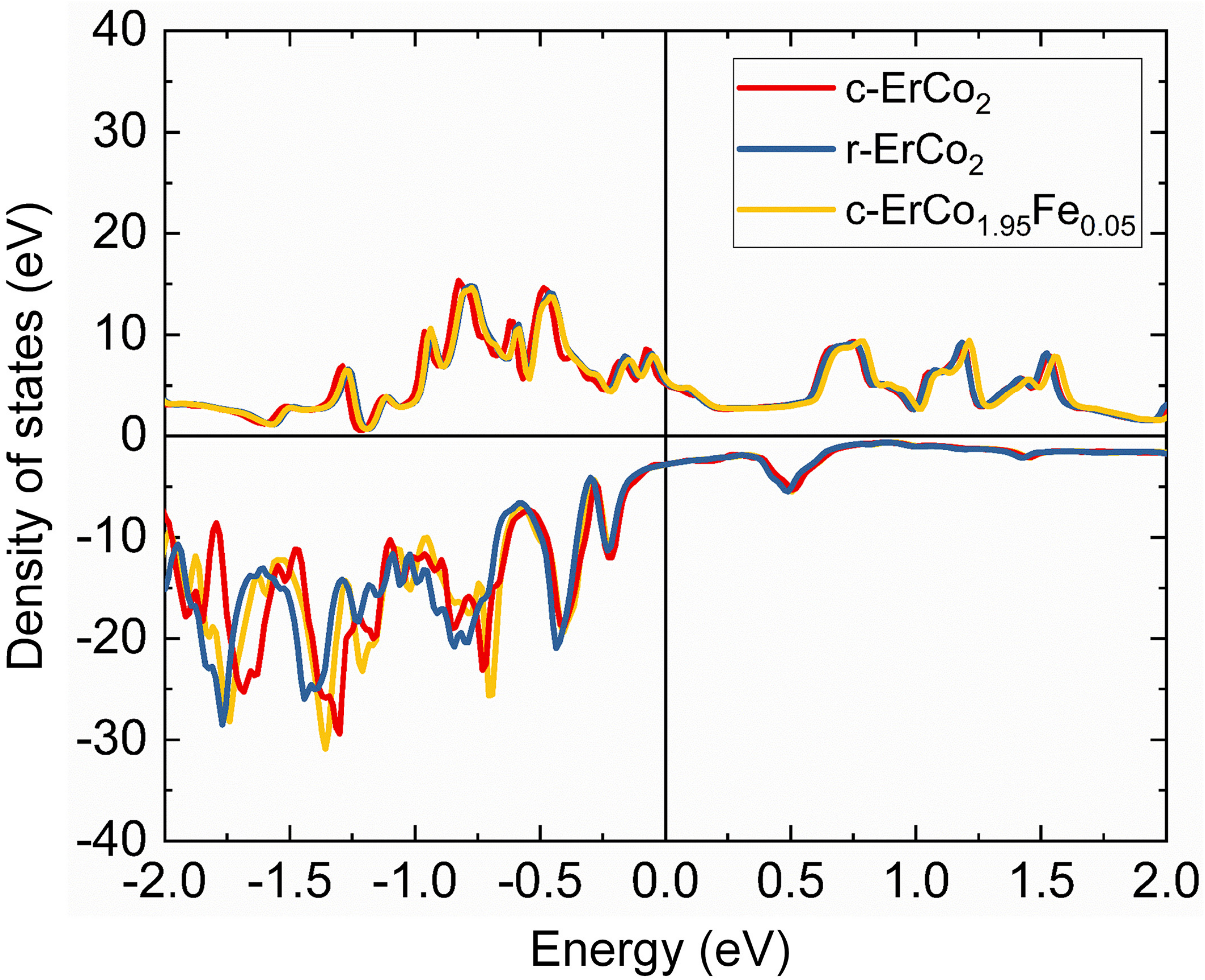

Supplementary Fig. 3. **First-principle calculations**. Density of states of ferrimagnetic cubic $ErCo_2$ (red), rhombohedral $ErCo_2$ (blue), and cubic $ErCo_{1.95}Fe_{0.05}$ compounds (yellow).

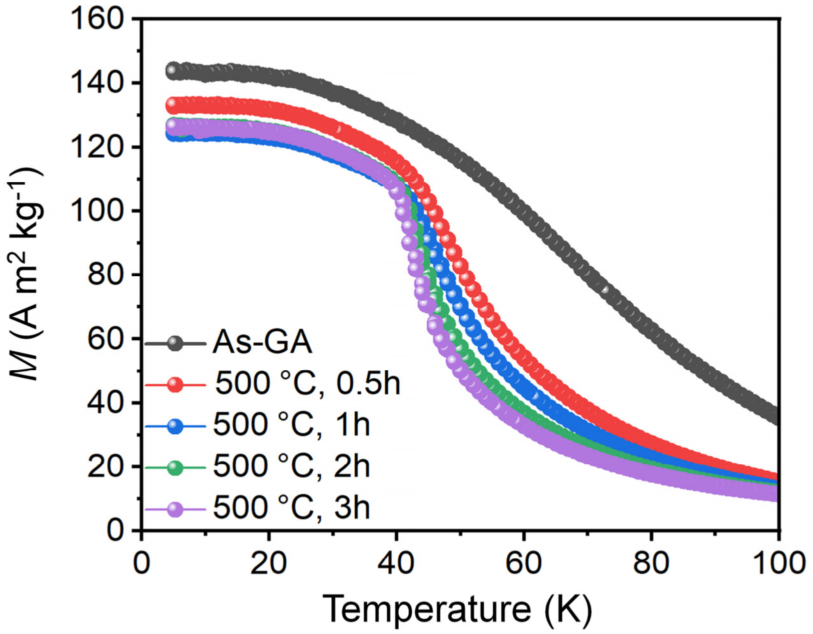

Supplementary Fig. 4. **Optimal magnetocaloric properties of as-gas atomised particles.** Temperature-dependent magnetisation *M* (T) curves for as-gas atomised (black) and post-annealed (coloured) $ErCo_{1.96}Fe_{0.04}$ powders at different annealing conditions. The selected colours of the curves correspond to different annealing conditions shown in the inset.

Supplementary Note 1: Determination of phase transition by X-ray diffraction pattern

The selected x-ray diffraction (XRD) patterns obtained at three temperatures (below, near, and above transition temperatures) for the $ErCo_2$, $ErCo_{1.96}Fe_{0.04}$, and $ErCo_{1.95}Fe_{0.05}$ alloys are shown in Fig. S5. In the XRD pattern for temperatures above the transition temperature, all of the characteristic peaks of these three samples can be indexed as the C15 phase (Fd-3m space group), except for one extra peak attributed to the Cu sample holder. However, when the temperature was decreased to below the transition temperature, the *440* peak of the cubic structure splits into two peaks, namely *220* and *208*, indicative of the characteristic features of the formation of the rhombohedral crystal structures for $ErCo_2$ and $ErCo_{1.96}Fe_{0.04}$ (Supplementary Fig. S5 a-b). These findings indicate that the crystal structure changed from cubic to rhombohedral (R-3m space group). By contrast, the crystal structure transition from cubic to rhombohedral was not observed for the $ErCo_{1.95}Fe_{0.05}$ alloy, revealing that an SOPT was realised in the $ErCo_{1.95}Fe_{0.05}$ alloy.

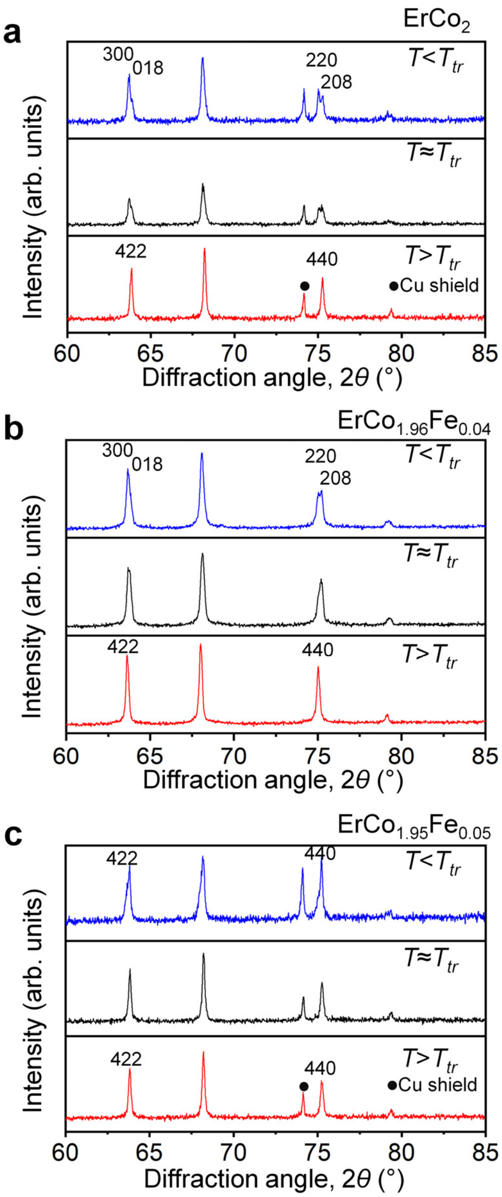

s

Supplementary Fig. 5: X-ray diffraction (XRD) pattern for **a** $ErCo_2$, **b** $ErCo_{1.96}Fe_{0.04}$, and **c** $ErCo_{1.95}Fe_{0.05}$ alloys at selected temperatures (*T*) above (red), around (black), and below transition temperature (T_{tr}) (blue).

Supplementary Note 2: Specific heat measurements

To investigate the nature of the phase transitions in $Er(Co_{1-x}Fe_x)_2$, we measured specific heat, C, for the $ErCo_2$, $ErCo_{1.96}Fe_{0.04}$ and $ErCo_{1.95}Fe_{0.05}$ samples using the thermal relaxation method. Generally, the relaxation method is not precise near the temperature of a first-order phase transition. Nevertheless, by tracking the sample temperature during the measurement, we could detect the first-order phase transition.

In the thermal relaxation method, the time evolution of the temperature difference between the sample and thermal sink, $\Delta T(t)$ is expressed by

$$\Delta T = \Delta T_{max}[1 - \exp{(-t/\tau)}] \quad (1)$$

because the heat balance is given by

$$P = C\frac{d(\Delta T)}{dt} + K_{\text{eff}}\Delta T. \quad (2)$$

A constant power, P, is supplied to the sample, $\tau = C/K_{\text{eff}}$, and K_{eff} is the effective thermal conductance of the sample. ΔT_{max} is the final temperature difference in the steady state, $\Delta T_{max} = P/K_{\text{eff}}$.

When the sample is at the first order phase transition temperature, a latent heat, Q, is generated during the temperature change of the relaxation method. Assuming that Q is constant during a single measurement, we can simply add the latent heat to eq. (2),

$$P = C\frac{d(\Delta T)}{dt} + K_{\text{eff}}\Delta T + Q. \quad (3)$$

In this case, $\Delta T_{max} = (P - Q)/K_{\text{eff}}$ $(\equiv \Delta T'_{max})$. Therefore, we can detect the first-order transition behaviour by evaluating whether or not ΔT_{max} is different from that in the normal condition without the latent heat Q.

In the present specific heat measurements, we performed the relaxation method three times at each temperature. For the three measurements, the sample temperature returned back to the same starting temperature after finishing previous measurement. Therefore, if the sample is at the first-order phase transition temperature, we should observe the latent heat only for the first measurement, while we do not observe it for the second and third measurements due to hysteretic nature of the first-order phase transition.
